# Circulating mitochondrial cell-free DNA dynamics in patients with mycobacterial pulmonary infections: Potential for a novel biomarker of disease

**DOI:** 10.3389/fimmu.2022.1040947

**Published:** 2022-11-15

**Authors:** Sheng-Wei Pan, Rehan R. Syed, Donald G. Catanzaro, Mei-Lin Ho, Chin-Chung Shu, Tsung-Yeh Tsai, Yen-Han Tseng, Jia-Yih Feng, Yuh-Min Chen, Wei-Juin Su, Antonino Catanzaro, Timothy C. Rodwell

**Affiliations:** ^1^ Department of Chest Medicine, Taipei Veterans General Hospital, Taipei, Taiwan; ^2^ School of Medicine, National Yang Ming Chiao Tung University, Taipei, Taiwan; ^3^ Division of Pulmonary, Critical Care and Sleep Medicine, Department of Medicine, University of California San Diego, La Jolla, CA, United States; ^4^ Division of Infectious Diseases and Global Public Health, University of California San Diego, La Jolla, CA, United States; ^5^ Department of Biological Sciences, University of Arkansas, Fayetteville, AR, United States; ^6^ Department of Chemistry, Soochow University, Taipei, Taiwan; ^7^ Department of Chemistry and Biochemistry, University of California San Diego, La Jolla, CA, United States; ^8^ Department of Internal Medicine, National Taiwan University Hospital, Taipei, Taiwan; ^9^ College of Medicine, National Taiwan University, Taipei, Taiwan; ^10^ Division of Chest Medicine, China Medical University Hospital, Taipei Branch, Taipei, Taiwan

**Keywords:** cell-free DNA, mitochondria, pulmonary tuberculosis, treatment response monitoring, biomarker

## Abstract

**Objectives:**

Human mitochondrial cell-free DNA (Mt-cfDNA) may serve as a useful biomarker for infectious processes. We investigated Mt-cfDNA dynamics in patients with pulmonary mycobacterial infections to determine if this novel biomarker could be used to differentiate disease states and severity.

**Methods:**

Patients with pulmonary tuberculosis (PTB), latent tuberculosis infection (LTBI), and nontuberculous mycobacterial-lung disease (NTM-LD) were enrolled at a tertiary care hospital in Taiwan between June 2018 and August 2021. Human Mt-cfDNA and nuclear-cfDNA (Nu-cfDNA) copy numbers were estimated by quantitative polymerase chain reaction. Variables associated with PTB and 2-month sputum culture-positivity, indicating poor treatment response, were assessed using logistic regression.

**Results:**

Among 97 patients with PTB, 64 with LTBI, and 51 with NTM-LD, Mt-cfDNA levels were higher in patients with PTB than in LTBI (p=0.001) or NTM-LD (p=0.006). In the *Mycobacterium tuberculosis*-infected population, Mt-cfDNA levels were highest in smear-positive PTB patients, followed by smear-negative PTB (p<0.001), and were lowest in LTBI persons (p=0.009). A Mt-cfDNA, but not Nu-cfDNA, level higher than the median helped differentiate culture-positive PTB from culture-negative PTB and LTBI (adjusted OR 2.430 [95% CI 1.139–5.186], p=0.022) and differentiate PTB from NTM-LD (adjusted OR 4.007 [1.382–12.031], p=0.011). Mt-cfDNA levels decreased after 2 months of treatment in PTB patients (p=0.010). A cutoff Mt-cfDNA level greater than 62.62 x 10^6^ copies/μL-plasma was associated with a 10-fold risk of 2-month culture-positivity (adjusted OR 9.691 [1.046–89.813], p=0.046).

**Conclusion:**

Elevated Mt-cfDNA levels were associated with PTB disease and failed sputum conversion at 2 months in PTB patients, and decreased after treatment.

## Introduction

An estimated 10 million people infected with *Mycobacterium tuberculosis* (MTB) develop active pulmonary tuberculosis disease (PTB) yearly (1), and up to one third of the population has latent tuberculosis infection (LTBI) at any time, with a 5–10% lifelong risk of developing PTB (2). LTBI, subclinical TB and active TB disease comprise the spectrum of MTB infection. The ability to identify individuals with PTB, differentiate LTBI from PTB and other mycobacterial infections, and to verify treatment success, is key both for individual patient outcomes and for preventing MTB transmission to others. Sputum culture positivity for MTB is one of the primary reference standards for TB diagnosis, just as the conversion from sputum positive to negative after 2 months of intensive-phase treatment is considered a reference indicator of therapeutic response. However, the slow growth of MTB cultures contributes to significant diagnostic delays, making evaluating sputum culture conversion both time-consuming and laborious. Easy-to-measure host inflammatory biomarkers that can differentiate PTB from other diagnoses or that can be used to support assessments of TB treatment response, would be transformative (3). Levels of c-reactive protein (CRP) and blood monocyte-to-lymphocyte ratio (MLR) may help to identify PTB in high-risk populations, and both decrease during treatment, but their changes are poorly predictive of sputum culture conversion at 2 months (3–8). Interferon-γ release assays (IGRA) measure interferon-γ response to MTB-specific antigens and are useful for identifying individuals with LTBI, however, they cannot be used to differentiate LTBI from PTB reliably, and are not useful for monitoring response to treatment (9, 10).

Cell-free DNA (cfDNA) is circulating fragmented DNA in blood that is actively secreted by living cells or released from dying cells and destroyed microorganisms and other pathogens (11, 12). Interest in cfDNA as a biomarker of altered physiologic and pathogenic states has prompted research into its diagnostic value in pregnancy and organ transplant monitoring and cancer diagnosis (13–15). Elevated levels of host-derived cfDNA can be also be predictive of a poor prognosis in infection (16–18). Both nuclear cfDNA (Nu-cfDNA) and mitochondrial cfDNA (Mt-cfDNA) are host-derived cfDNA, with the former originated from the nucleus and the later from mitochondria distributed throughout the cytoplasm in a cell. They can be released by activated inflammatory cells and only Mt-cfDNA can be released by platelets during infection (19, 20). Studies have shown that both plasma Nu-cfDNA and Mt-cfDNA levels correlate with disease severity and mortality in patients with sepsis (18, 21, 22). Prior research has also demonstrated that among HIV-positive individuals, plasma Mt-cfDNA levels were higher in those with TB and severe inflammation than in those without disease (23). *Wiens et al.* reported that macrophages infected with more virulent MTB released more mitochondrial DNA into the cytosol, suggesting mitochondrial DNA could be a biomarker indicating innate immune responses in TB (24). Studies have also demonstrated that MTB can cause mitochondrial damage in macrophages, leading to the release of mitochondrial DNA (25). Yet, it remains unclear if the release of Mt-cfDNA from inflammatory cells into the extracellular space, presumably resulting in high circulating Mt-cfDNA level, is related to burden of MTB nor if observed levels are different for LTBI or PTB and other pulmonary mycobacterial diseases. It is also not yet known if elevated Mt-cfDNA levels at PTB diagnosis reverts to baseline after PTB disease is effectively treated.

We hypothesized that plasma Mt-cfDNA is associated with MTB bacterial burden and inflammatory response in TB. In order to explore these ideas we a) examined the association between plasma Mt-cfDNA levels in patients with and without culture-positive PTB; b) evaluated changes in plasma Mt-cfDNA level during PTB treatment and c) evaluated the potential predictive value of Mt-cfDNA levels on subsequent failure of sputum conversion after two months of treatment. Finally, we measured levels of Mt-cfDNA in supernatants from a cell line stimulated with MTB early secretory antigenic target (ESAT)-6 antigen to develop potential supporting evidence for the causative role of MTB on Mt-cfDNA release from macrophages.

## Methods

### Study design and enrollment

This prospective observational study was conducted at the Taipei Veterans General Hospital in Taipei City, Taiwan, from June 2018 to August 2021. The cohort was composed of patients with LTBI, PTB, and nontuberculous mycobacterial-lung disease (NTM-LD). Adult outpatients with PTB were enrolled if 1) their respiratory samples were culture-positive for MTB or 2) they were culture-negative but a lung biopsy showed compatible pathologic findings and tested positive for the MTB-specific *IS6110* gene (6). Asymptomatic persons with a history of close contact in the past six months with another PTB patient (prior to enrollment) were included if they had normal chest radiography and IGRA-positivity, indicating LTBI (26). As comparators, patients with NTM-LD were enrolled if they had positive NTM cultures from respiratory samples and met the NTM-LD diagnostic criteria for active disease (27, 28). The exclusion criteria were 1) HIV infection in PTB patients tested at enrollment and in NTM-LD patients assessed by reviewing claim data, 2) concomitant active cancer, and 3) mixed MTB and NTM-LD infection. Written informed consents were obtained from participants (Taipei Veterans General Hospital Institutional Review Board Nos. 2017-12-001C, 2018-10-017A, 2019-07-003C, 2020-07-009AC, 2021-01-010BC).

### Blood sampling for cfDNA test

Peripheral blood samples were obtained from all participants at baseline before treatment. In patients with PTB, blood tests were performed again after two months of standard intensive-phase anti-TB therapy. For plasma preparation, 8–10 mL of whole blood was collected with K2-EDTA Vacutainer tubes (BD, Franklin Lakes, NJ, USA) and centrifuged for 10 minutes at 1500 rpm within 2 hours of collection (29). Using the QIAamp DNA Blood Mini Kit (Qiagen, Hilden, Germany), cfDNA was extracted from a 400 µL plasma sample with an elution volume of 50 µL and stored in a microcentrifuge tube at −80°C until real-time quantitative polymerase chain reaction (qPCR) was performed in batches (30, 31).

### Real-time qPCR with absolute quantification

To estimate the Mt-cfDNA and Nu-cfDNA copy numbers in plasma samples, the levels of a noncoding human mitochondrial genome region (hMito) and a nuclear DNA, human beta2 microglobulin (hB2M), were measured by qPCR (20). To calculate the absolute copy number of the target genes from the cycle threshold (Ct) value, a reference 5-point standard curve was constructed in duplicate on each plate with known copies of hMito and hB2M amplicons as previously reported (20). The levels of hMito and hB2M in plasma were expressed as copy numbers per μL of the original plasma sample (copies/μL plasma) after adjustment for the dilution factor of 1.25 (see **Supplement Appendix 1** for details).

### Clinical data collection and the outcome

Patient clinical data were collected at baseline for patients with PTB and NTM-LD and at 2 months among those who underwent anti-TB treatment. Data collected included body mass index (BMI), smoking status, medical comorbidities, radiographic score and findings (32), and sputum acid-fast bacilli cultures results and smear grades. Laboratory data including CRP and complete blood count with lymphocytes, monocytes, and platelet counts were also recorded. Persons with LTBI and patients with NTM-LD were confirmed to have no microbiologic evidence of PTB during a follow-up period of at least 3 months. Successful sputum culture conversion from positive to negative after two months of treatment was the outcome measure in patients with culture-positive PTB.

### Measuring cfDNA in macrophages stimulated by MTB antigens

To try and establish a more direct link between MTB infection and the release of Mt-cfDNA and Nu-cfDNA from macrophages, we also examined the supernatant of monocytes stimulated with MTB antigens. Using a human leukemic cell line (THP-1) (TIB-202, ATCC, Manassas, VA, USA), THP-1 cells were cultured and treated with phorbol 12-myristate 13-acetate (PMA, 20 ng/ml) (P8139, Sigma-Aldrich, St. Louis, MO, USA) for 24 hours, followed by PMA removal and rest for 72 hours (33, 34). Differentiated cells were then stimulated by MTB-specific recombinant ESAT-6 protein (Recombinant Protein Reference Standard, NR-49424) (BEI Resources, Manassas, VA, USA) (34) to mimic a TB infection. After ESAT-6 protein was used to stimulate cells at concentrations of 0, 0.1, 1.0, and 2.0 µg/mL for 24 hours, culture supernatants were harvested for qPCR targeting Mt-cfDNA and Nu-cfDNA with absolute quantification.

### Statistical analysis

The Mann–Whitney *U-*test and Student’s *t*-test were used to compare continuous variables between PTB, LTB and NTM-LD groups. A paired *t*-test was performed to compare the cfDNA levels before and after treatment for PTB. The distribution of a categorical variables between groups were compared by the χ^2^ test and Fisher’s exact test. Linear correlations between cfDNA levels and interested continuous variables were evaluated using Pearson correlation analysis. Logistic regression analysis was used to identify factors associated with culture-positive PTB and sputum conversion. Odds ratios (ORs) and 95% confidence intervals (CIs) were calculated using logistic regression. All variables with a *p*-value less than or equal to 0.05 in a univariate analysis were entered in a multivariable analysis of independent factors. Areas under the receiver operating characteristic (ROC) curves (AUCs) for variables of interest were calculated and if the lower boundary of a 95% CI for the AUC was above 0.5 the optimal cut-off value was determined (35, 36). Analyses were completed using GraphPad Prism version 6.0 (GraphPad Software Inc, CA, USA) and SPSS version 18.0 (SPSS Inc, IL, USA).

## Results

### Characteristics of participants

**Figure 1:**
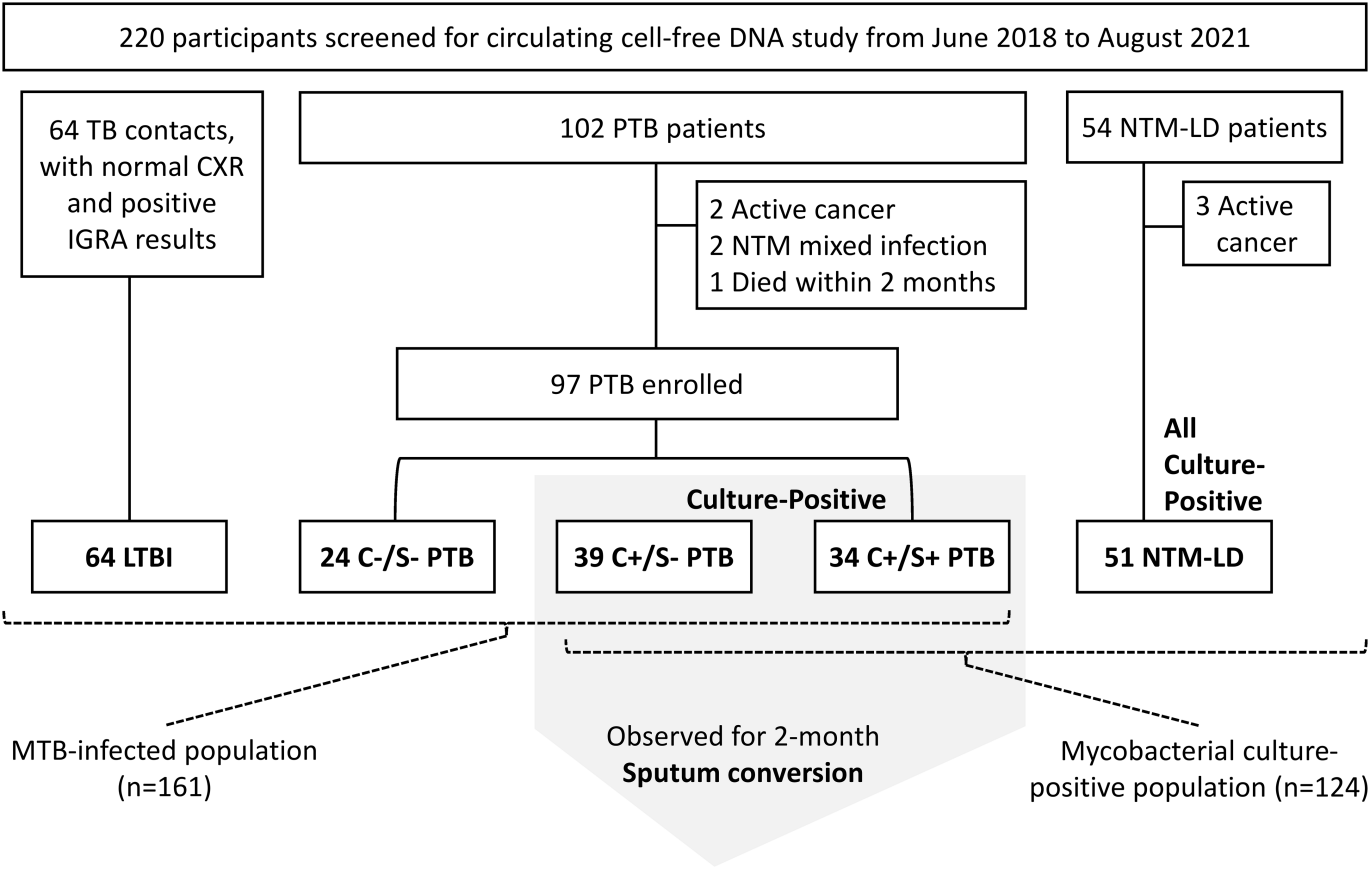
Flow chart of participant enrollment. C+, culture-positive; C-, culture-negative; S+, smear-positive; S-, smear-negative; LTBI, latent tuberculosis infection; NTM-LD, nontuberculous mycobacterial lung disease; PTB, pulmonary tuberculosis.

Sixty-four TB contacts with LTBI, 97 patients with PTB, and 51 with NTM-LD were enrolled over the 3-year study period (**Figure 1**). Among PTB patients, 73 were culture-positive while 24 were culture-negative. Of the culture-positive cases, 34 (46.6%) were smear-positive and 12 (16.4%) had isoniazid-resistant MTB isolates. No isolates were rifampicin resistant. The platelet count, monocyte count, MLR, and CRP levels in the PTB group were all higher than those in the LTBI group (all p<0.05) but similar to those in the NTM-LD group (**Table 1**). Although sputum smear grades were similar between the PTB and NTM-LD groups, the rates of hemoptysis, bilateral lung disease, and radiographic scores were higher in the NTM-LD group.

**Table 1:** Demographic and clinical characteristics of patients with latent tuberculosis infection (LTBI), pulmonary tuberculosis (PTB), and nontuberculous mycobacterial lung disease (NTM-LD) (N=212).

Variables	LTBI (n=64)	PTB (n=97)	NTM-LD (n=51)
Age, years	63.3 ± 18.1	60.9 ± 19.4	64.7 ± 13.7
Male Sex	29 (45%)	58 (60%)	17 (33%)**
BMI (kg/m^2^)	23.5 ± 3.5*	22.0 ± 3.3	19.9 ± 3.2***
Smoking status			
Current smoker	8 (13%)	15 (15%)	2 (4%)
Ex-smoker	9 (14%)	23 (24%)	9 (18%)
Comorbidity			
Prior TB history	4 (6%)	11 (11%)	3 (6%)
Diabetes mellitus	9 (14%)	21 (22%)	4 (8%)*
Cancer history	5 (8%)	9 (9%)	5 (10%)
COPD	2 (3%)	2 (2%)	5 (10%)*
Blood cell count			
Platelet count (K/μL) (N=210)	226.0 ± 61.7*	259.3 ± 107.1	236.8 ± 73.2
Monocyte count (/μL) (N=208)	489.4 ± 186.0**	578.3 ± 252.2	519.4 ± 194.7
Lymphocyte count (/μL) (N=208)	1787.6 ± 620.7***	1414.6 ± 529.1	1302.7 ± 568.3 (n=41)
Monocyte-to-lymphocyte ratio (N=208)	0.29 ± 0.11***	0.48 ± 0.32	0.57 ± 0.75
C-reactive protein (mg/L) (N=174)	0.24 ± 0.47***	1.20 ± 1.96	1.55 ± 2.51
Symptoms			
Hemoptysis	0	4 (4%)	12 (24%)**
Fever	0	4 (4%)	3 (6%)
Culture positive for Mycobacterium	–	73 (75%)	51 (100%)
Smear-positive (N=124)	–	34 (35%)	23 (45%)
Sputum smear grade (N=124)	–	0 [0–1]	0 [0-1]
Bilateral lung disease	–	34(35%)	40 (78%)***
Radiographic score	–	2 [2–4]	5 [4-9]***

Continuous and categorical data are expressed as mean ± standard deviation (SD) or median with interquartile range [IQR] and number (%)

*indicates p < 0.05 as compared to PTB group; **p < 0.01; ***p < 0.001.

BMI, body mass index; COPD, chronic obstructive pulmonary disease.

### Mt-cfDNA and Nu-cfDNA levels in groups

**Figure 2:**
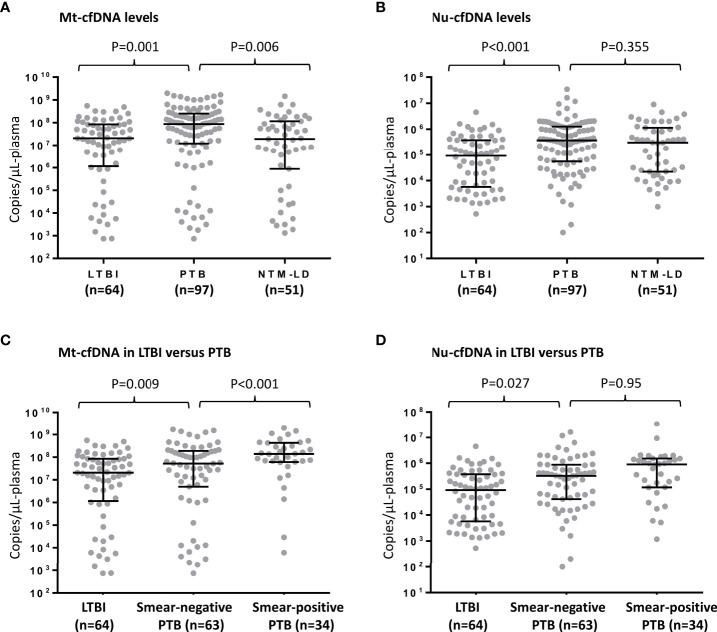
Human mitochondrial cell-free DNA (Mt-cfDNA) **(A)** and nuclear cell-free DNA (Nu-cfDNA) **(B)** levels in persons with latent tuberculosis infection (LTBI), pulmonary tuberculosis (PTB) and nontuberculous mycobacterial lung disease (NTM-LD). The levels of Mt-cfDNA **(C)** and Nu-cfDNA **(D)** in LTBI, smear-negative PTB (n=63, including 24 pathology-confirmed and 39 culture-positive cases) and smear-positive PTB subgroups. Data are presented as medians with interquartile ranges, and with each dot representing a value.

Median levels of Mt-cfDNA and Nu-cfDNA were 45.44 x10^6^ copies/μL-plasma (interquartile range [IQR] 4.52 x10^6^–157.73 x10^6^) and 22.25 x10^4^ copies/μL-plasma (IQR 2.17 x10^4^–90.45 x10^4^), respectively. Mt-cfDNA levels were higher in the PTB group (87.22 x10^6^ copies/μL-plasma [IQR 12.20 x10^6^–252.24 x10^6^]) than in the LTBI group (20.63 x10^6^ copies/μL-plasma [IQR 1.15 x10^6^–84.68 x10^6^]) (p=0.001, **Figure 2A**). Similarly, Nu-cfDNA levels were higher in the PTB group (37.01 x10^4^ copies/μL-plasma [IQR 5.51 x10^4^–126.44 x10^4^]) than in the LTBI group (9.18 x10^4^ copies/μL-plasma [IQR 0.57 x10^4^–37.53 x10^4^]) (p<0.001, **Figure 2B**). The median Mt-cfDNA level in the PTB group was also higher than that in the NTM-LD group (19.32 x10^6^ copies/μL-plasma [IQR 0.89 x10^6^–115.07 x10^6^]) (p=0.006, **Figure 2A**) but the Nu-cfDNA was not (30.27 x10^4^ copies/μL-plasma [IQR 2.22 x10^4^–113.94 x10^4^] in NTM-LD) (p=0.355, **Figure 2B**). Among patients infected with MTB, including PTB and LTBI but excluding NTM-LD, the level of Mt-cfDNA was higher in smear-positive PTB patients than in smear-negative PTB patients (p<0.001), but higher in smear-negative PTB patients than in persons with LTBI (p=0.009) (**Figure 2C**). This trend of association was not evident with Nu-cfDNA levels (**Figure 2D**). For patients with NTM-LD, Mt-cfDNA levels were not different between smear-positive and smear-negative cases (p=0.523). Overall, and even only among the PTB patients, Mt-cfDNA level was correlated with Nu-cfDNA, platelet count, monocyte count, and CRP (all p<0.01) and was not associated with age, gender, BMI, lymphocyte count, or MLR (see **Supplement Table S1**).

### Factors associated with culture-positive PTB

In the MTB-infected cohort consisting of persons with LTBI and PTB but not NTM-LD (**Figure 1**), factors associated with culture-positive PTB in the multivariable analysis included BMI, platelet count, MLR, and Mt-cfDNA level higher than the median value of 45.44 x10^6^ copies/μL-plasma (adjusted OR 2.430, 95% CI 1.139–5.186, p=0.022) (**Table 2**). In the mycobacterial culture-positive cohort consisting of culture-positive PTB and NTM-LD patients, the independent factors associated with culture-positive PTB were sex, platelet count, radiographic score, and Mt-cfDNA level higher than 45.44 x10^6^ copies/μL-plasma (adjusted OR 4.007, 95% CI 1.382–12.031, p=0.011) (**Table 2**).

**Table 2:** Logistic regression model of factors associated with the diagnosis of culture-positive pulmonary tuberculosis (PTB).

Variable	Univariate		Multivariate, backward elimination	
	Crude OR (95% CI)	*p* value	Adjusted OR (95% CI)	*p* value
In MTB-infected population (N=161)				
Age (year)	1.003 (0.986–1.019)	0.764		
Male sex	1.953 (1.037–3.680)	0.038	1.333 (0.602–2.948)	0.478
BMI (kg/m^2^)	0.870 (0.788–0.959)	0.005	0.887 (0.789–0.996)	0.043
Platelet count (K/ul)	1.006 (1.003–1.010)	0.001	1.007 (1.002–1.012)	0.007
Monocyte count (/μL) (N=160)	1.003 (1.001–1.005)	0.002	-^a^	
Lymphocyte count (/μL) (N=160)	0.999 (0.998–0.999)	< 0.001	-^a^	
Monocyte-to-lymphocyte ratio (N=160)	1.056 (1.032–1.080)	< 0.001	1.058 (1.030–1.086)^a^	< 0.001
C-reactive protein (mg/L) (N=145)	2.287 (1.415–3.696)	0.001	-^b^	
Nu-cfDNA (10^4^ copies/μL-plasma)	1.000 (1.000–1.000)	0.099		
Mt-cfDNA (10^6^ copies/μL-plasma)	1.002 (1.001–1.004)	0.007	–	
Mt-cfDNA > 45.5 x10^6^ copies/μL-plasma^c^	2.379 (1.256–4.503)	0.008	2.430 (1.139–5.186)	0.022
In culture-positive PTB and NTM-LD cases (N=124)				
Age (year)	0.992 (0.972–1.013)	0.462		
Male sex	3.407 (1.607–7.224)	0.001	5.060 (1.752–14.612)	0.003
BMI (kg/m2)	1.195 (1.058–1.351)	0.004	1.064 (0.905–1.251)	0.450
Platelet count (K/ul) (N=122)	1.004 (1.000–1.008)	0.048	1.010 (1.004–1.016)	0.002
Monocyte count (/μL) (N=121)	1.002 (1.000–1.004)	0.059		
Lymphocyte count (/μL) (N=121)	1.000 (1.000–1.001)	0.469		
Monocyte-to-Lymphocyte ratio (N=121)	0.998 (0.991–1.005)	0.584		
C-reactive protein (mg/L) (N=145)	0.977 (0.810–1.179)	0.808		
Sputum smear grade	1.187 (0.891–1.582)	0.241		
Bilateral lung disease	0.203 (0.090–0.458)	< 0.001	0.723 (0.181–2.882)	0.645
Radiographic score	0.637 (0.530–0.767)	< 0.001	0.528 (0.374–0.747)	< 0.001
Nu-cfDNA (10^4^ copies/μL-plasma)	1.000 (1.000–1.000)	0.356		
Mt-cfDNA (10^6^ copies/μL-plasma)	1.002 (1.000–1.003)	0.035	–	
Mt-cfDNA > 45.5 x10^6^ copies/μL-plasma^c^	2.802 (1.339–5.864)	0.006	4.007 (1.382–12.031)	0.011

a In this model, lymphocyte and monocyte counts were not entered since they were highly correlated with monocyte-to-lymphocyte ratio.

b In a sensitivity analysis with the model adjusting for CRP level (n=145), Mt-cfDNA levels above 45.5 x10^6^ copies/μL-plasma remained significantly correlated to culture-positive PTB (adjusted OR 2.310, 95% CI 1.028–5.189, p=0.043).

c This cut-off value was determined by the median levels of Mt-cfDNA in the total population.

BMI, body mass index; Mt-cfDNA, mitochondria cell-free DNA; NTM-LD, nontuberculous mycobacterium (NTM) lung disease; Nu-cfDNA, nuclear cell-free DNA.

### Sputum conversion and Mt-cfDNA changes in the PTB group

**Figure 3:**
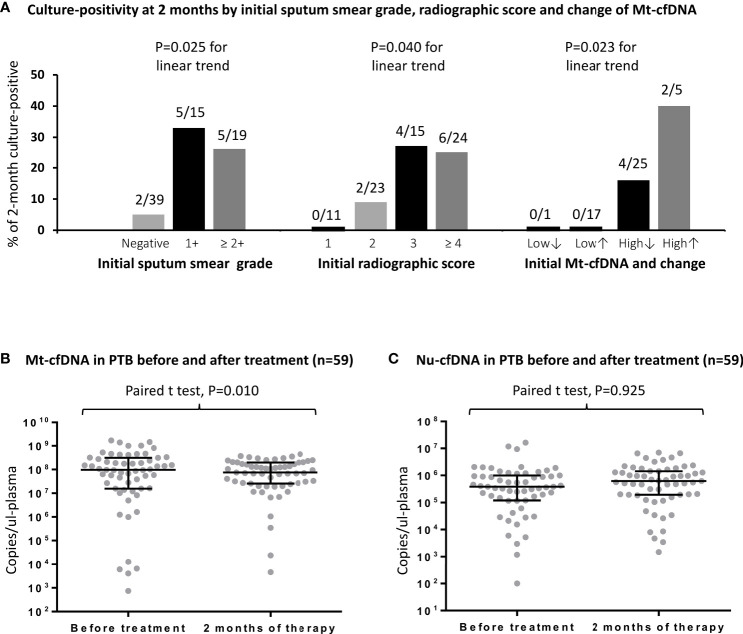
The rates and numbers (n/N) of 2-month culture-positivity in PTB patients are shown stratified by initial sputum smear grade, initial radiographic score, and dynamic change of Mt-cfDNA levels **(A)**.The levels of human mitochondrial cell-free DNA (Mt-cfDNA) **(B)** and nuclear cell-free DNA (Nu-cfDNA) **(C)** at baseline and at 2 months of therapy in patients with pulmonary tuberculosis (PTB). Here, Mt-cfDNA levels at 2 months in the 59 patients with PTB were normally distributed and a paired *t* test was used. Data are presented as medians with interquartile ranges, and with each dot representing a value. Low↓, low Mt-cfDNA level (<62.62 x 10^6^ copies/μL-plasma) at baseline with a further decrease at 2 months; Low↑, low Mt-cfDNA level at baseline with a subsequent increase at 2 months; High↓, high Mt-cfDNA level (> 62.62 x 10^6^ copies/μL-plasma) at baseline with a subsequent decrease at 2 months; High↑, high Mt-cfDNA level at baseline with a further increase at 2 months.

Twelve (16.4%) of the 73 initially culture-positive PTB patients were still culture-positive for MTB after 2 months of treatments. Two-month culture-positivity was associated with sputum smear grade and radiographic score at enrollment (**Figure 3A**). In ROC curve analyses, the AUC for smear grade and radiographic scores to discriminate persistent culture-positivity after 2 months of anti-TB therapy were 0.716 (95% CI 0.569–0.862) and 0.736 (95% CI 0.586–0.885), respectively, while that for Mt-cfDNA level was 0.658 (95% CI 0.507–0.810). The AUCs for Nu-cfDNA level, platelet count, monocyte count, MLR, and CPR were 0.634 (95% CI 0.480–0.788), 0.536 (95% CI 0.325–0.746), 0.663 (95% CI 0.488–0.839), 0.582 (95% CI 0.416–0.748), and 0.643 (95% CI 0.490–0.795), respectively, all with 95% CI lower bounds below 0.5. Using the optimal cutoff value of 62.62 x 10^6^ copies/μL-plasma to dichotomize Mt-cfDNA level, the sensitivity, specificity, positive and negative predictive values of high Mt-cfDNA to predict two-month culture-positivity were 91.67%, 49.18%, 26.19% and 96.77%, respectively. An Mt-cfDNA level above this level was associated with a 10-fold risk of persistent culture-positivity (crude OR 10.645, 95% CI 1.293–87.607, p=0.028). The results were similar after adjusting for smear grade and radiographic score (adjusted OR 9.691, 95% CI 1.046–89.813, p=0.046) (**Table 3**).

**Table 3:** Logistic regression model of factors associated with persistent culture-positive at 2 months of treatment in patients with PTB (N=73).

Variable	Univariate		Multivariate, backward elimination	
	Crude OR (95% CI)	*p* value	Adjusted OR (95% CI)	*p* value
Age (year)	1.014 (0.982–1.047)	0.391		
Male sex	3.472 (0.700–17.226)	0.128		
BMI (kg/m^2^)	0.986 (0.815–1.194)	0.888		
Smoking status				
Never smoker	1 (reference)			
Current smoker	1.689 (0.417–6.843)	0.463		
Ex-smoker	1.583 (0.269–9.320)	0.611		
Blood cell count				
Platelet count (K/μL)	1.003 (0.998–1.008)	0.297		
Monocyte count (/μL)	1.001 (0.999–1.003)	0.465		
Lymphocyte count (/μL)	1.000 (0.998–1.001)	0.664		
Monocyte-to-lymphocyte ratio	1.012 (0.996–1.028)	0.128		
C-reactive protein (mg/L) (N=68)	1.108 (0.855–1.435)	0.439		
Sputum smear grade	1.494 (1.004–2.223)	0.048	1.140 (0.668–1.944)	0.631
INH resistance strain	1.543 (0.279–8.532)	0.619		
Bilateral lung disease	2.158 (0.614–7.589)	0.230		
Radiographic score	1.521 (1.137–2.035)	0.005	1.427 (1.008–2.021)	0.045
Nu-cfDNA (10^4^ copies/μL-plasma)	1.000 (1.000–1.000)	0.982		
Mt-cfDNA (10^6^ copies/μL-plasma)	1.002 (1.000–1.002)	0.230	–	
Mt-cfDNA > 62.6 x10^6^ copies/μL-plasma^a^	10.645 (1.293–87.607)	0.028	9.691 (1.046–89.813)	0.046

a Optimal cut-off value determined using receiver operating characteristic curve analysis.

BMI, body mass index; Mt-cfDNA, mitochondria cell-free DNA; Nu-cfDNA, nuclear cell-free DNA.

Among the PTB patients with follow-up blood tests (n=59, including 48 with initial culture-positivity), the median Mt-cfDNA level was lower after 2 months of treatment when compared to baseline level (75.31 x10^6^ copies/μL-plasma [IQR 25.53 x10^6^–197.93 x10^6^] versus 97.78 x10^6^ copies/μL-plasma [IQR 15.72 x10^6^–314.22 x10^6^], p=0.010 using paired *t*-test) but the Nu-cfDNA level was not statistically different (**Figure 3B, C**). We also examined Mt-cfDNA changes in 48 PTB patients with initial culture-positivity and used Mt-cfDNA dynamics to stratify patients (**Figure 3A**). In those patients with initially low levels of Mt-cfDNA (less than 62.62 x 10^6^ copies/μL-plasma), all were culture negative at two months regardless of subsequent changes in Mt-cfDNA levels, while in those with initially high levels that subsequently decreased (starting above 62.62 x 10^6^ copies/μL-plasma), 16% (4/25) were culture positive at two months, and in those with initially high and subsequently increasing levels of Mt-cfDNA, 40% (2/5) were still culture positive at two months (p=0.023 for trend) (**Table 4**).

**Table 4:** Characteristics of pulmonary tuberculosis (PTB) patients stratified by Mt-cfDNA dynamic change at 2 months of treatment (N=59).

	initially low Mt-cfDNA, decreased at 2 months (n=4)	initially low Mt-cfDNA, increased at 2 months (n=19)	initially high Mt-cfDNA, decreased at 2 months (n=28)	initially high Mt-cfDNA, increased at 2 months (n=8)	P value for trend^a^
Variables
Initial Mt-cfDNA (10^6^ copies/μL-plasma)	33.7 ± 24.7	14.6 ± 17.9	465.6 ± 451.3	129.8 ± 70.7	0.023
Change at 2M (10^6^ copies/μL-plasma)	-19.6 ± 20.4	+75.7 ± 74.3	-354.1 ± 421.8	+126.1 ± 132.7	0.228
Age (years)	54.7 ± 24.2	59.8 ± 19.7	60.1 ± 22.6	69.7 ± 18.1	0.273
Male sex	2 (50%)	12 (63%)	15 (54%)	6 (75%)	0.742
BMI (kg/m^2^)	20.0 ± 2.8	20.5 ± 2.5	21.5 ± 3.5	22.8 ± 4.0	0.062
Blood cell count					
Platelet count (K/ul)	246.8 ± 78.2	256.8 ± 84.5	267.7 ± 122.2	221.2 ± 106.2	0.745
Monocyte count (/μL)	432.7 ± 188.8	597.6 ± 341.5	597.5 ± 235.1	648.8 ± 229.3	0.318
Lymphocyte count (/μL)	1128.7 ± 510.9	1189.7 ± 394.0	1434.7 ± 530.7	1207.7 ± 612.3 (n=41)	0.363
Monocyte-to-lymphocyte ratio	0.45 ± 0.32	0.53 ± 0.21	0.46 ± 0.22	0.82 ± 0.81	0.174
C-reactive protein (mg/L) (N=55)	0.59 ± 0.50	1.70 ± 2.60	1.06 ± 1.47	0.47 ± 0.68	0.342
In initial culture-positive PTB patients (N=48)					
Before treatment sputum smear grade	3	0 [0-0.25]	0 [0-1.5]	1 [0.5-3]	0.571
Before treatment smear-positive (n/N)	1/1	4/17 (24%)	12/25 (48%)	4/5 (80%)	0.090
**2-month persistent culture-positive (n/N)**	**0/1**	**0/17**	**4/25 (16%)**	**2/5 (40%)**	**0.023**
Bilateral lung disease at baseline	1 (25%)	4 (21%)	11 (39%)	4 (50%)	0.168
Radiographic score at baseline	2 [2–2.75]	2 [2–4]	3 [1.25–3.75]	3 [2–7.25]	0.182

Continuous and categorical data are expressed as mean ± standard deviation (SD) or median with [IQR] and number (%).

BMI, body mass index; Mt-cfDNA, mitochondria cell-free DNA; mo., months.

a Associations were assessed by Pearson’s correlation test for continuous variables and by χ^2^ test for linear trends for binary variables.The bold values are the subgroup names according to Mt-cfDNA change at 2 months.

### Mt-cfDNA in supernatant from stimulated monocytes

**Figure 4:**
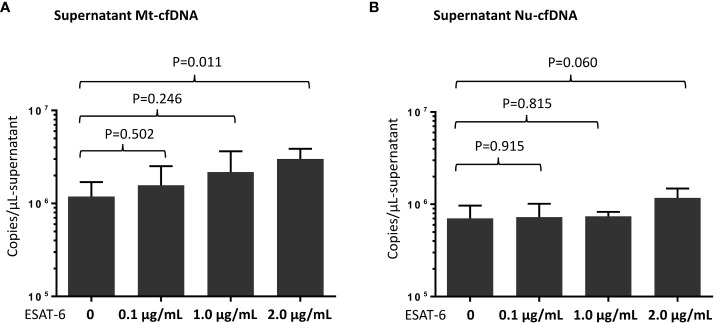
Measurement of mitochondrial cell-free DNA (Mt-cfDNA) **(A)** and nuclear cell-free DNA (Nu-cfDNA) **(B)** in supernatants from phorbol 12-myristate 13-acetate (PMA)-treated human leukemic cell culture after stimulation by recombinant 6 kDa early secretory antigenic target (ESAT-6) protein (at concentrations of 0, 0.1, 1.0 and 2.0 µg/mL). Data are presented as the mean ± standard deviation, and n=4 at every concentration level.

We quantified levels of Mt-cfDNA and Nu-cfDNA in supernatants from THP-1 cells (primary monocyte differentiated macrophages) stimulated with MTB ESAT-6 antigens. We observed a significantly higher mean Mt-cfDNA level (3.00 x10^6^ ± 0.87 x10^6^ copies/μL-plasma) in the supernatant after stimulation with high-dose (2.0 µg/mL) ESAT-6 protein than in the supernatants with no MTB antigen stimulation (1.18 x10^6^ ± 0.50 x10^6^ copies/μL-plasma) (p=0.011). But there was no significant difference in Nu-cfDNA level between the MTB antigen stimulated and non-stimulated samples (**Figure 4A, B**).

## Discussion

In our study of patients with MTB and NTM infections we found that Mt-cfDNA levels were elevated in patients with PTB as compared to those with LTBI and NTM-LD, despite the greater clinical severity of disease among the NTM-LD patients. Notably, plasma Mt-cfDNA was positively associated with inflammatory markers and smear-positivity (a proxy for bacterial burden) in PTB patients, indicating a potential association of this biomarker with disease severity. For patients with culture positive PTB, high and increasing levels of Mt-cfDNA also appeared to have some potential for predicting persistent culture-positivity after 2 months of treatment, but more importantly, low Mt-cfDNA levels at start of treatment was highly predictive of culture negativity after two months of treatment. Furthermore, while evaluating the plausibility of our proposed linkage between plasma Mt-cfDNA and MTB infection, we observed stimulation of THP-1 cells with MTB antigen ESAT-6 is associated with Mt-cfDNA release into the extracellular space of inflammatory cells, supporting the hypothesis for the origin of the increased plasma Mt-cfDNA levels in PTB.

Identifying individuals with infectious PTB and monitoring treatment response remain critical aspects of TB control worldwide. Of the currently available blood biomarkers for this purpose, CRP performs better than symptom-based methods as a screening tool for PTB in people living with HIV (4), but neither its baseline level nor change over time is predictive of MTB sputum conversion, which despite its limitations, is still considered the reference standard for treatment success (5). Elevated monocyte and platelet counts are correlated with inflammation in PTB and trend down after treatment, but they cannot reliably predict treatment outcomes (37–39). MLR may have utility in identifying TB disease in HIV-infected children and for differentiating active TB from LTBI in the general population (6–8), but persistently high MLRs have not been found to associate 2-month culture-positivity (7, 8). The present study, while limited in size and scope, indicates that Mt-cfDNA levels are elevated in individuals with PTB compared to those with LTBI which reinforces the potential of Mt-cfDNA as a novel biomarker for differentiating PTB from LTBI, possibly in conjunction with existing assays such as the IGRAs. In PTB patients, Mt-cfDNA level was positively correlated with CRP, monocyte count, and platelet count, and was higher in smear-positive patients than in smear-negative patients, suggesting that Mt-cfDNA is correlated with both inflammatory processes and bacterial burden. Our findings are supported by prior research showing elevated Mt-cfDNA to be associated with TB-related immune reconstitution inflammatory syndrome and interleukin-18, a proinflammatory cytokine, in HIV-infected patients (23). From a clinical standpoint, when excluding MTB smear-positive patients who can more easily be diagnosed with PTB using existing diagnostics, Mt-cfDNA level was also statistically different between smear-negative PTB patients and the contacts with LTBI, suggesting a potential role for Mt-cfDNA for differentiating low-bacterial-burden PTB from IGRA positive LTBI patients. However, the presence of outliers with low Mt-cfDNA levels in the smear-negative PTB subgroup suggests that Mt-cfDNA alone might not have sufficient sensitivity in the clinical setting to differentiate all smear-negative PTB from LTBI. However, it may nonetheless play complementary role in the medical decision making model when evaluating these patients. Based on the totality of our results, we believe that human origin Mt-cfDNA has the potential to be used as an indicator of the clinical stage and severity of MTB infection, reflecting the clinical spectrum of MTB infection from LTBI to smear-negative and smear-positive PTB, and its use as a standalone or complementary biomarker in the evaluation of patients with suspected PTB and LTBI warrants further investigation.

Importantly, our study demonstrates that Mt-cfDNA may also have some utility in treatment monitoring for patients with PTB. A high baseline Mt-cfDNA level was found to be moderately predictive of 2-month culture-positivity in PTB patients and it was observed that Mt-cfDNA levels decline after 2 months of treatment. Our observed rate of persistent culture-positivity of 16.4% at 2 months is in line with previous reports (35, 40). Notably, using changes in Mt-cfDNA to stratify patients, we noted that the rate of persistent 2-month culture-positivity was highest in the PTB subgroup with initially high Mt-cfDNA and subsequently increasing levels at 2 months while the rates were lower in other subgroups, with a statistically significant trend. We hypothesize that Mt-cfDNA could be an integrated surrogate marker for bacterial load and inflammation in PTB, which are important factors associated with decreased rates of on-treatment culture-conversion (3, 41, 42). Similarly, decreased Mt-cfDNA levels reflect decreased bacterial load and reduced inflammation following treatment, which could have clinical relevance in indicating successful treatment. To this end, while the exact cause of increased circulating Mt-cfDNA levels is likely complex and multidimensional, our proof-of-concept study suggests the potential value of Mt-cfDNA monitoring the treatment response of PTB.

Human cfDNA originates from cell apoptosis, neutrophil extracellular traps, and the death of inflammatory cells in response to microbes, as well as from active cellular secretion through extracellular vehicles (12). In comparison to Nu-cfDNA, Mt-cfDNA is a thousand-fold higher in copy numbers because every cell, except red blood cells, contains abundant mitochondria with DNA. We found Mt-cfDNA levels better correlate with culture-positive PTB and predict 2-month culture-positivity than Nu-cfDNA. These findings may be mechanistically supported by the relationship we observed between ESAT-6 stimulation and Mt-cfDNA levels in supernatants from activated THP-1 cells. However, human Mt-cfDNA may be elevated by various physical or psychiatric stresses and pathologic insults (17, 43, 44). To investigate the specificity of Mt-cfDNA as a biomarker of PTB, we also included patients with NTM-LD in the study and found that high Mt-cfDNA facilitated the differentiation of culture-positive PTB from NTM-LD. Interestingly, although the NTM-LD group had higher rates of hemoptysis and higher radiographic scores indicating more severe disease among the NTM patients, elevated Mt-cfDNA levels still differentiated PTB from NTM-LD suggesting the possibility of a unique association between PTB disease and Mt-cfDNA level that goes beyond lung pathology. Using MTB strains with different virulence to infect bone marrow-derived macrophages, *Wiens et al.* reported the strain-dependent infection-related release of mitochondrial DNA into the cytosol (24). Because the levels of several inflammatory markers and bacterial load were similar in the PTB and NTM-LD groups, the differentially higher level of Mt-cfDNA in PTB patients may indicate a specific role for MTB in causing the release of Mt-cfDNA that deserves further investigation.

The present study has some limitations. First, the timing of laboratory testing during the intensive phase of treatment in PTB patients was limited to only two time points and the number of cases eligible for persistence culture-positivity assessment was relatively small. Large-scale studies are warranted to validate the association between dynamic changes in Mt-cfDNA during follow-up and bacterial burden during and after tuberculosis treatment. Second, the enrolled participants were clinically stable HIV-uninfected outpatients at a single institute, so the generalizability of our findings to other populations of different conditions on diverse treatment regimens remains unknown. Further studies on the performance of Mt-cfDNA in differentiating mycobacterial diagnosis and predicting outcomes among PTB and NTM-LD patients with HIV co-infection are needed. In addition, confounders such as diabetes severity and antihyperglycemic medications might affect the treatment outcome in PTB patients but were not available for analysis (45). Our study is also limited by the lack of a healthy control group. In one recently published study, the authors disclosed that patients with multidrug-resistant TB had increased mitochondrial DNA copy numbers in blood leukocytes when compared to healthy individuals, and proposed this finding was a result of oxidative stress in TB patients (46), which may be one of the plausible mechanisms explaining the elevated extracellular plasma Mt-cfDNA levels in our patients with PTB. Further investigations incorporating healthy controls may help confirm the potential role of Mt-cfDNA in differentiating the spectrum ranging from an uninfected population, to LTBI and PTB. Finally, the plasma samples were not double centrifuged and, thus, the level of cfDNA may have been overestimated (31). However, our results should be robust because, using the same protocol, all intergroup and post-treatment comparisons consistently demonstrated that high levels of Mt-cfDNA were associated with PTB-related inflammation and bacterial burden. Nevertheless, the potential impact of differences in cfDNA measurement protocols among different studies should be acknowledged.

In conclusion, we found that Mt-cfDNA level was correlated with inflammatory markers and smear-positivity in PTB. Importantly, Mt-cfDNA, but not Nu-cfDNA, was associated with culture-positive PTB, was associated with sputum culture-positivity at 2 months of therapy, and decreased after treatment. Further studies are warranted to evaluate the potential complementary role of Mt-DNA in PTB identification and treatment response monitoring.

## Data availability statement

The raw data supporting the conclusions of this article will be made available by the authors, without undue reservation. Requests to access these datasets should be directed to Sheng-Wei Pan, sanweipan@gmail.com.

## Ethics statement

The studies involving human participants were reviewed and approved by Taipei Veterans General Hospital Institutional Review Board Nos. 2017-12-001C, 2018-10-017A, 2019-07-003C, 2020-07-009AC, 2021-01-010BC. The patients/participants provided their written informed consent to participate in this study.

## Author contributions

S-WP, T-YT, J-YF, and W-JS collected data. All authors performed data analysis. S-WP, RS, DC, M-LH, and C-CS wrote the manuscript. J-YF and TR provided critical revisions for the paper. S-WP and J-YF were the guarantors of the paper, taking responsibility for the integrity of the work as a whole, from inception to published article. All authors contributed to the article and approved the submitted version.

## Funding

S-WP was funded by grants from Taipei Veterans General Hospital [V110C-040] and the Ministry of Science and Technology [107-2314-B-075-057, 108-2314-B-075-001, 109-2314-B-075-094, 110-2314-B-075-077, 111-2314-B-075-059] in Taiwan. TR, DC and AC were funded by NIH NIAID grant # R01AI137681.

## Acknowledgments

The authors thank the Medical Science and Technology Building of the Taipei Veterans General Hospital and the Stein clinical research building of the University of California, San Diego, for providing research facilities. The authors also would like to thank Dr. Peter G Chiles for providing help in the experiment consultation.

## Conflict of interest

The authors declare that the research was conducted in the absence of any commercial or financial relationships that could be construed as a potential conflict of interest.

## Publisher’s note

All claims expressed in this article are solely those of the authors and do not necessarily represent those of their affiliated organizations, or those of the publisher, the editors and the reviewers. Any product that may be evaluated in this article, or claim that may be made by its manufacturer, is not guaranteed or endorsed by the publisher.
