# Supplementary material for: Circulating mitochondrial cell-free DNA dynamics in patients with mycobacterial pulmonary infections: Potential for a novel biomarker of disease
Source: Front Immunol. 2022 Nov 15;13:1040947. doi: 10.3389/fimmu.2022.1040947 (PMC9709461; doi:10.3389/fimmu.2022.1040947)
Supplement: Supplementary file 1 [file DataSheet_1.doc]

Original article

Article Title: **Circulating mitochondrial cell-free DNA dynamics in patients with mycobacterial pulmonary infections: Potential for a novel biomarker of disease**

Authors: **Sheng-Wei Pan^a,b,c^, Rehan R Syed^d^, Donald G Catanzaro^e^, Mei-Lin Ho^f,g^, Chin-Chung Shu^h,i*^, Tsung-Yeh Tsai^a,b^, Yen-Han Tseng^a,b^, Jia-Yih Feng^a,b,*^, Yuh-Min Chen^a,b^, Wei-Juin Su^j^, Antonino Catanzaro^c^, Timothy C Rodwell^c^**

Institution:

^a^Department of Chest Medicine, Taipei Veterans General Hospital, Taipei, Taiwan

^b^School of Medicine, National Yang Ming Chiao Tung University, Taipei, Taiwan

^c^Division of Pulmonary, Critical Care and Sleep Medicine, Department of Medicine, University of California San Diego, La Jolla, California, USA.

^d^Division of Infectious Diseases and Global Public Health, University of California San Diego, La Jolla, California, USA.

^e^Department of Biological Sciences, University of Arkansas, Fayetteville, Arkansas, USA

^f^Department of Chemistry, Soochow University, Taipei, Taiwan.

^g^Department of Chemistry and Biochemistry, University of California San Diego, La Jolla, California, USA.

^h^Department of Internal Medicine, National Taiwan University Hospital, Taipei, Taiwan.

^i^College of Medicine, National Taiwan University, Taipei, Taiwan.

^j^Division of Chest Medicine, China Medical University Hospital, Taipei Branch, Taipei, Taiwan.

* Drs. Chin-Chung Shu and Jia-Yih Feng contributed equally to this work

**Running Title:** **Mitochondrial cfDNA in TB patients**

**Keywords:** cell-free DNA; mitochondria, monitor, pulmonary tuberculosis

**The corresponding author:**

**Jia-Yih Feng, MD., PhD.**

Department of Chest Medicine, Taipei Veterans General Hospital

No. 201, Sec. 2, Shih-Pai Rd., Taipei, Taiwan. E-mail: peterofeng@gmail.com

**Supplementary material**

**Appendix 1. Cell-free DNA primers and qPCR conditions**

The primers for the human mitochondrial genome region (hMito) gene were hMito_F3= 5’ CACTTTCCACACAGACATCA-3’ and hMito_R3 =5’ TGGTTAGGCTGGTGTTAGGG-3’ (128-bp product). The primers for the human beta2 microglobulin (hB2M) gene were hB2M_F1= 5’ TGTTCCTGCTGGGTAGCTCT-3’ and hB2M_R1= 5’ CCTCCATGATGCTGCTTACA-3’ (188-bp) [1]. For real-time quantitative polymerase chain reaction (qPCR) , 2 μL template DNA samples were added to the duplicate wells, which contained 200 mM forward and 200 mM reverse primers (0.8 μL), 10 μL 2X SYBR Green PCR Master Mix, 2 μL QN ROX Reference Dye, and 5.2 μL of PCR-grade water. The qPCR cycling program performed a starting hold at 95 °C for 1 minute, followed by 40 cycles consisting of denaturation at 95 °C for 3 seconds, annealing at 60 °C for 40 seconds, and extension at 70 °C for 30 seconds, with final holds at 95 °C for 15 seconds and 62 °C for 1 minute (Applied Biosystems 7500 Fast PCR System, Thermo Fisher Scientific, Waltham, MA, USA). To calculate the absolute copy number of the target genes from the cycle threshold (Ct) value, a reference 5-point standard curve was constructed in duplicate on each plate with known copies of hMito and hB2M amplicons as previously reported [1]. The levels of hMito and hB2M in plasma were expressed as copy numbers per μL of the original plasma sample (copies/μL plasma) after adjustment for the dilution factor of 1.25 (plasma cfDNA extraction: 400 µL plasma eluting to 50 µL; qPCR dilution: 2 µL cfDNA sample in a total reaction volume of 20 µL).

**Reference** [1]. Rosa HS, Ajaz S, Gnudi L, AN. M. A case for measuring both cellular and cell-free mitochondrial DNA as a disease biomarker in human blood. FASEB J 2020; 34:12278-88.

**Table S1**. Associations between Mt-cfDNA and clinical parameters in whole study population (overall) and pulmonary tuberculosis (PTB) group

|  | Overall | | | PTB group | | |
| --- | --- | --- | --- | --- | --- | --- |
| Variables | N | Pearson’s r | P value | N | Pearson’s r | P value |
| Nu-cfDNA | 212 | 0.591 | < 0.001 | 97 | 0.624 | < 0.001 |
| Age, years | 212 | -0.130 | 0.059 | 97 | -0.120 | 0.242 |
| BMI (kg/m^2^) | 212 | -0.069 | 0.320 | 97 | -0.092 | 0.371 |
| Platelet count (K/μL)) | 210 | 0.309 | < 0.001 | 97 | 0.320 | 0.001 |
| Monocyte count (/μL) | 208 | 0.220 | 0.001 | 97 | 0.273 | 0.007 |
| Lymphocyte count (/μL) | 208 | -0.036 | 0.609 | 97 | 0.037 | 0.717 |
| Monocyte-to-Lymphocyte ratio | 208 | 0.054 | 0.435 | 97 | 0.116 | 0.259 |
| C-reactive protein (mg/L) | 174 | 0.266 | < 0.001 | 92 | 0.357 | < 0.001 |
| Radiographic score | - | - | - | 97 | 0.204 | 0.045 |

BMI indicates body mass index; Mt-cfDNA, mitochondria cell-free DNA; Nu-cfDNA, nuclear cell-free DNA.
